# The anterior hypothalamic area: a hub for convergence of innate and learned threat responding

**DOI:** 10.1007/s00429-026-03085-w

**Published:** 2026-02-26

**Authors:** Cailey M. Coletta, Brenton Laing

**Affiliations:** Department of BioMolecular Sciences, University of Mississippi, Oxford, MS 38673 USA

**Keywords:** Anterior hypothalamic area, AHA, AHN, survival behavior, threat responding, circuits, cytoarchitecture, tracing, defense

## Abstract

The anterior hypothalamic area (AHA) has been implicated in a diverse array of functions ranging from defensive behavior selection to autonomic control. However, new methods that allow systematic characterization of the anatomical organization, connectivity, activity patterns, and molecular diversity have revealed the AHA as a hub for the convergence of innate and learned threat responding. Threat information is relayed to the AHA by synaptic inputs and circulating factors to convey information relevant for the regulation of organ system physiology and behavior. This structure is a critical hub for the regulation of survival behaviors but is relatively understudied compared to other hypothalamic structures. This review focuses on recent advances in dissecting the AHA that have overcome historical challenges that limit understanding of the AHA’s role in basic brain circuitry and health disorders.

## Introduction

The hypothalamus is a highly conserved brain region that is extraordinarily rich in molecular diversity that controls behavioral and physiological regulation of survival. While some subregions of the hypothalamus have been extensively studied, the anterior hypothalamic area (AHA) remains a black box in computation despite being identified in the medial hypothalamic defensive system decades ago (Canteras 2002). The AHA is a hub for the convergence of synaptic inputs and circulating factors that convey information about innate and learned threats in the environment with synaptic output that targets regions to coordinate the state and action of the animal. This review will synthesize what is known about the synaptic connectivity of the AHA, as well as the respondent physiological and behavioral states that are generated by AHA output.

## Cytoarchitecture of the AHA

**Fig. 1:**
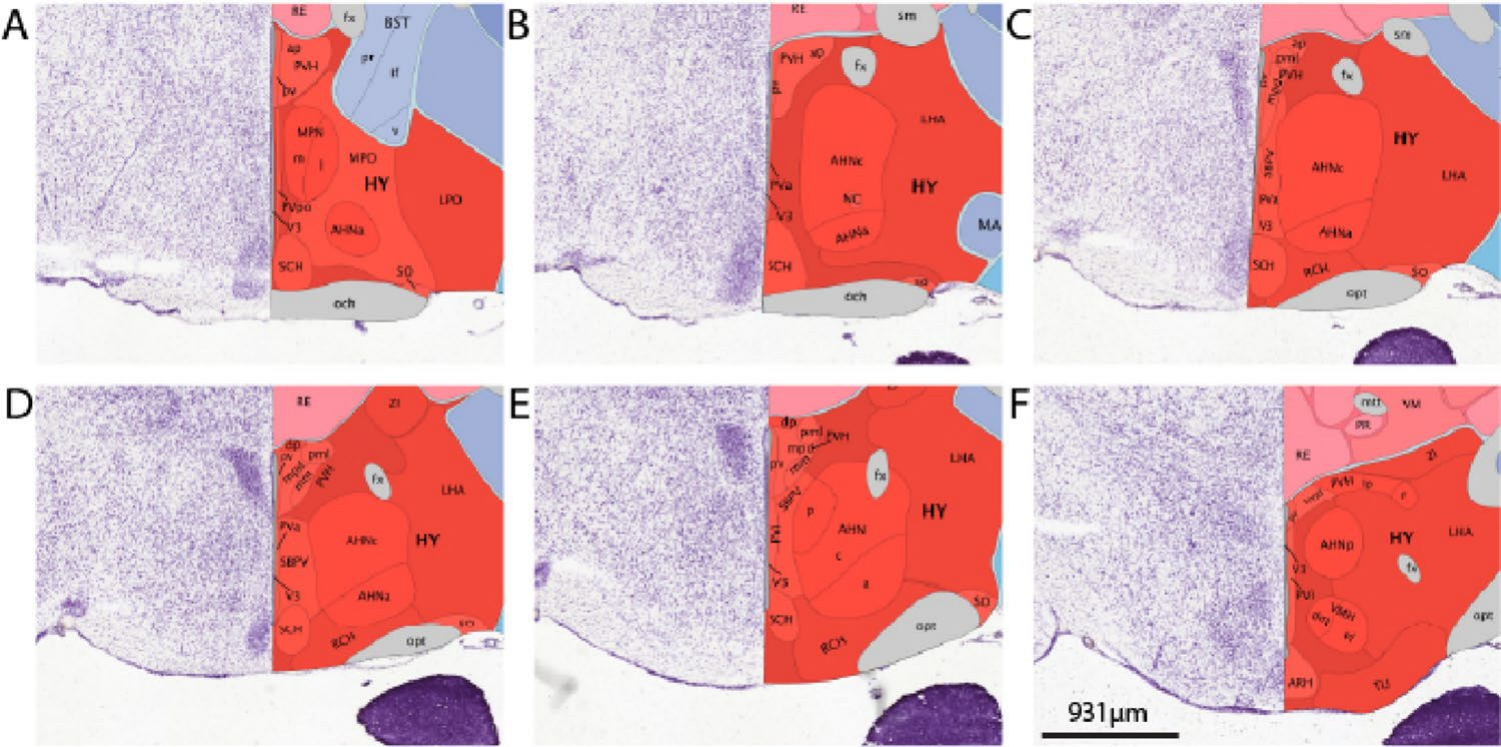
Representative images of AHA parcellation on coronal mouse sections. Images visualized from anterior to posterior sections through the AHA adapted from the Allen Brain coronal reference atlas (https://mouse.brain-map.org/experiment/thumbnails/100048576?image_type=atlas). **A** The anterior segment of the AHA, which is very challenging to distinguish from the medial preoptic area by Nissl staining. **B** The AHA segment which contains the central region surrounding the nucleus circularis (NC) as well as a segment of the anterior parcel. **C** The central AHA and anterior AHA, which is easy to distinguish from the paraventricular hypothalamus (PVH) and suprachiasmatic nucleus (SCH), but challenging to distinguish from the lateral hypothalamic area (LHA). **D** The central and anterior parcels of the AHA. **E** Visualization of all three AHA parcels, with the anterior segment situated ventrolateral and posterior segment situated dorsomedial to the central segment (respectively). **F** The most posterior appearance of the AHA, which can be distinguished from the ventromedial hypothalamus (VMH) and PVH by Nissl staining, but is challenging to visualize a clear boundary with the LHA

The AHA is well conserved across mammalian species from the mouse to the rat, cat, and monkey (Saper et al. 1978). It contains predominantly medium to small sized neurons arranged in three condensations (anterior, central, posterior). Notably, in the judgment of Saper et al. the central cell aggregation often referred to as the ‘anterior hypothalamic nucleus’ is not sufficiently distinct to warrant a special designation (Saper et al. 1978). In keeping with that judgment, we refer to the region as the anterior hypothalamic area here. Paxinos and Franklin draw the mouse AHA as a hypothalamic region that exists in anterior/posterior (AP) coordinates between − 0.34 mm and − 1.34 mm and between medial/lateral (ML) coordinates of ± 0.25 mm and ± 0.75 mm. The structure is situated as a tube that is more ventrally located in the anterior coordinates, between − 5.3 mm and − 5.6 mm dorsal/ventral (DV), and more dorsally located in the posterior coordinates, between − 5.0 mm and − 5.3 mm. Work by Saper, Swanson, and Cowan elegantly described the AHA with classic cellular staining methodology (Saper et al. 1979). At the anterior segment (Fig. 1A–E) the boundary that distinguishes the AHA from the preoptic area and lateral hypothalamus is not easily distinguishable by nuclei staining with DAPI or Nissl staining. The cell area of cells in the preoptic/anterior hypothalamus is reported to be larger in males than females in C57BL6/J mice, though there was no relationship between cell size and estrous stage (Brown et al. 1999). However, DAPI and Nissl staining does effectively mark distinguishable boundaries between the AHA and the paraventricular nucleus, the supraoptic nucleus, and the suprachiasmatic nucleus (Saper et al. 1978). Thus, the cytoarchitectural approach is very useful for identifying AHA cells that are adjacent to these well-defined structures. The AHA structure’s center, denoted the anterior hypothalamic area central region (AHC), is identified between the − 0.7 to −1.15 AP where it surrounds the nucleus circularis (Fig. 1B–E), also referred to as the circular nucleus. The central portion of the AHA reportedly contains smaller cells than the anterior portion (Saper et al. 1978). Situated posterior to the AHC is the anterior hypothalamic area posterior region (AHP) (Fig. 1E, F). The AHP consists of scattered cells of similar size and staining characteristics to the surrounding areas that are separated from the dorsomedial and ventromedial nuclei by relatively low density areas which are visible with DAPI staining. These sex differences were not observed in littermates of *SF-1* gene disrupted wild-types or 129SvEv adults. This finding was extended by a report that males have a greater area of cells expressing estrogen receptor beta immunoreactivity than females (Wolfe et al. 2005).

To date, the AHA is reported to contain neurons that are marked by vesicular GABAergic transporters (AHA^VGAT^) (Laing et al. 2023), glutamatergic transporters (AHA^VGLUT2^), parvalbumin (AHA^PV^), and CaMKIIa (AHA^CaMKIIa^) (Hong et al. 2025). There is not evidence that the reference definitions of AHA regions can be parcellated by molecularly defined cell types. Multiple reports suggest the AHA is approximately 90% AHA^VGAT7,8^. In the AHA, *CaMKIIa* marks subsets of the approximately 51% AHA^VGAT^ populations marked by GABA immunostaining (Hong et al. 2025). Of note, the proportion of *CamKIIa* cells that are not **GABAergic** and density of the population appear to mark a distinct population of non-GABA/non-Vglut2 cells (Hong et al. 2025), though more observations with different approaches would strengthen this given the possibility of leaky expression caused by *CaMKIIa* promoter driven reporter expression rather than a cre-lox system. AHA^PV^ neuron expression is reported to be dependent on thyroid hormone signaling during development (Harder et al. 2018). These neurons were demonstrated to be glutamatergic by in situ hybridization, co-localization of immunostaining for parvalbumin with adeno-associated viral driven expression of cre-dependent green fluorescent protein in Vglut2-Cre but not in Vgat-Cre mice, and channelrhodopsin2 assisted circuit mapping with excitatory post-synaptic currents that were abolished by AMPA receptor blocker CNQX (Laing et al. 2023). No inhibitory post-synaptic currents were detected in that experiment. There is evidence by immunostaining that AHA^VGLUT2^ neurons express GABA (Hong et al. 2025), so it remains a possibility that GABA is synthesized. It is also possible that non-specific staining makes cell counting of neurotransmitter immunostaining difficult to interpret. It is also inconclusive if there is release of GABA, which would likely depend on vesicular GABA transporter expression. Of note, co-localization between *Vglut2* and *Vgat* was not observed in the AHA in intersectional studies that labeled both populations (Xu et al. 2022). However, this study did not report any Vglut2 expression in the AHA so perhaps there were too few to notice. It is possible but currently unknown if these neurons co-release some peptide, particularly at the high frequency firing rate that could cause sufficient calcium influx to support large dense core vesicle release.

## Stimulus dependent AHA activity changes

Early FOS in situ hybridization studies showed that swimming and restraint stress are reported to increase activation of the AHA (Cullinan et al. 1995), though it is not possible to parcellate the sub-regions based on the images or data provided in that report. Further studies demonstrated activation of the AHA in rodents by cat exposure and predatory context at the rostral, medial, and caudal levels (Cezario et al. 2008), though the extent of FOS expression was higher during actual predator exposure than the predatory context. Notably, all three sections included here are level 24–26 in the Swanson brain atlas which would correspond to the AHC and AHP. Thus, it is not possible to parcellate the effects on AHC from AHP based on this dataset. In addition, the poor temporal resolution of FOS (e.g. delay from stimulus presentation to expression) limits the interpretability of these studies between stimuli and neuronal activity patterns. Still, the intriguing foundation they provide is a strong justification for further research. This is consistent with rostral and caudal AHA FOS expression following rat exposure compared to a toy (Martinez et al. 2008). These histological analyses showing the effect of threatening stimuli exposure on increased AHA activity have been extended to strategies that acquire real-time neuronal activity measurements using miniature microscopy. In GCaMP expressing AHA neurons, we observed learned associations of threat information that significantly increase activation of neurons in the AHA following shock-induced fear conditioning during the unconditioned shock exposure and next-day conditioned tone exposure (Laing et al. 2025). Thus, using both miniature microscopy and FOS approaches, it is evident that conditioned stimuli evoke less activation than the unconditioned stimuli used to condition them. Together this indicates that AHA activity is generally tuned to activate during innate threat exposure but can become conditioned to incorporate broader contextual cues and conditioned stimuli in the animal’s environment.

It is evident from reports across numerous groups that the activity pattern changes of the entire nucleus are generally reflected in subpopulations. Live predatory exposure significantly increased GCaMP fluorescence levels in AHA^VGLUT2^, AHA^GAD2^, and AHA^CaMKIIa^ neuronal populations when in close proximity (Hong et al. 2025). Notably, AHA^GAD2^ and AHA^CaMKIIa^ neurons reached their peak activity during escape initiation, whereas AHA^VGLUT2^ neurons, despite an initial increase, reached their peak during the escape. AHA^VGAT^ activity also is reported to increase during risk assessment, object exploration, and thermal nociception as evidenced by fiber photometry recordings (Xie et al. 2022). This is consistent with defense associated increases in activation of AHA^VGAT^ neurons that is evident during attack from a CD1 mouse but not a social encounter (Xie et al. 2022), as well as theta rhythmicity that is decreased during aversive interactions and increased during affiliative interactions. These bidirectional effects in molecularly defined inhibitory neurons may be related to observations that conditioned shock fear exposure increased the firing rate of a cluster of AHC neurons which was inhibited by sucrose intake in control recordings (Mitra et al. 2016), though those electrophysiological recordings lacked any information about the molecular identity of the neurons. In line with these findings, we found that activity of AHA^VGAT^ neurons is predictive of the presence of rat predatory threat using machine modeling (Laing et al. 2023), though this may be a heterogenous population that adds variability to analysis by parametric statistics. We have also reported significant activation of molecularly defined glutamatergic AHA^PV^ neurons during innate threat exposure. More data is needed that tests the effects of stressors or learned escape, for example by the Flight Upon Grid Approach assay (Torossian et al. 2025).One report indicates that AHA^PV^ neurons exhibit changes in activity during heat exposure, with approximately two-thirds being heat excited and one-third being heat inhibited (Mittag et al. 2013). It is possible that these neurons are temperature sensors like those in the medial preoptic area (Machado and Saper 2022) and could conceivably be tied into fever or ambient temperature changes. However, this work requires further testing to fully understand the implications (Table 1).

Activity in the AHA is influenced by numerous circulating factors. The region of the AHA that lies on the boundary of the periventricular area appears to have dense expression of androgen receptor in mice in a tear-drop nucleus shape, which appears far less pronounced in rat (Jahan et al. 2015), but the function of these receptors is unclear. Electrophysiological analysis of activity changes in response to tail-vein injection of cytokines shows that IL-1β and IL-2 decreases activity of AHA neurons, with IL-1β dependent changes being localized towards the lateral edge and IL-2 dependent changes being more broadly distributed, while IL-6 showed weaker effects (Bartholomew and Hoffman 1993). The IL-1 dependent AHA activity changes are suspected to be indirect because IL-1 receptor expression is scant in the AHA but rich in the hippocampus, anterodorsal nucleus of the thalamus, and raphe. The IL-2 effects may be dependent on other hypothalamic regions or as direct effectors. Of note, intravenous tetanus toxin injection results in an increase of FOS expression in the AHA (Korneva et al. 2000). Glutamate microinjection to the central amygdaloid nucleus increases activity of angiotensin II sensitive neurons in the AHA, which can be blocked with losartan (Hagiwara et al. 2005), which indicates that angiotensin II modulates AHA activity patterns. Thyrotropin releasing hormone has mixed effects on AHA parvalbumin neuron activity with approximately 48% excited and 19% inhibited, while very few neurons (3/17) show a response to angiotensin II^17^. Thus, it is likely that the predominant angiotensin responsive neurons in the AHA are not AHA^PV^ neurons. Based on the representative image in that manuscript, it is not possible to identify any relationship between AHA parcellation and responses of cells.

## Afferent projections to the AHA

**Fig. 2:**
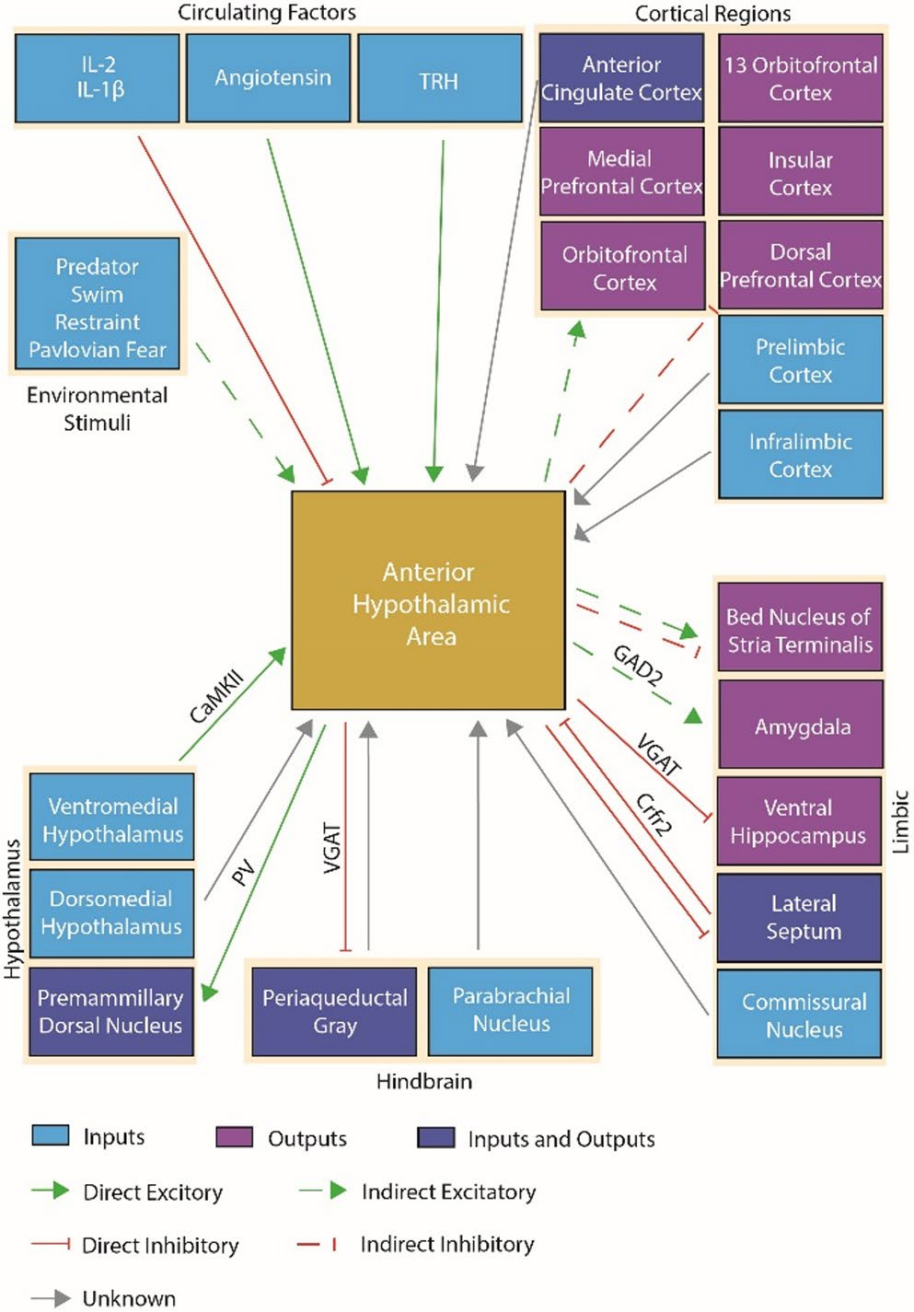
Schematic of anterior hypothalamic area (AHA) inputs and outputs. Direct synaptic inputs from the ventral hippocampus, lateral septum, and ventromedial hypothalamus have been shown to regulate activity patterns of the AHA by the mechanisms shown. Other regions, such as the dorsomedial hypothalamus, medial amygdala, commissural nucleus, and parabrachial nucleus, prelimbic cortex, and infralimbic have been demonstrated as efferent to the AHA by tracing methods but the neurotransmitter is not known. Circulating factors, such as TRH and angiotensin, have been shown to have a direct effect on AHA neurons while peripherally delivered cytokines have effect on AHA activity via an unknown mechanism. Evidence of monosynaptic output from the AHA to the lateral septum, premammillary dorsal nucleus, and periaqueductal gray have been demonstrated while indirect activity changes have been observed in cortical and limbic effectors. Solid lines indicate direct synaptic connections or effects, while dotted lines represent indirect or unknown connection routes evidenced by activity changes

The AHA is a hub for converging synaptic inputs (Fig. 2). Rabies retrograde tracing of AHA^VGAT^ neurons showed projections from the lateral septum, medial preoptic area, the paraventricular hypothalamus, the supraoptic nucleus, the ventromedial hypothalamus, the dorsomedial hypothalamus, the posterior hypothalamus, the dorsal and ventral mammillary nucleus, the lateral parabrachial nucleus, the paraventricular thalamus, and the medial amygdala (Xie et al. 2022). Much of this evidence is corroborated by other reports, with the notable discrepancy of the absence of the ventral hippocampus. Ventral hippocampal projections to the AHA convey contextual information about the presence of predators that contributes to goal-directed behavior that is dependent on escape opportunity in the environment (Bang et al. 2022a). It is unclear if this is because of the regional targeting of the AHA, if it is due to a discrepancy in the cells targeted, or some other source of variation. However, it is evident that excitatory ventromedial hypothalamic (VMH) neurons send projections to the AHA (Laing et al. 2025). Cholera-toxin B tracing of the AHA revealed Crfr2 neurons in the lateral septum send projections to the AHA (Anthony et al. 2014). This was further validated using channelrhodopsin2 assisted circuit mapping which demonstrates GABAergic release from these neurons. The pericommissural nucleus, which is activated by predatory threat (Canteras and Goto 1999a), is reported to send projections to the AHA but the effects of those projections on AHA activity patterns is incompletely understood (Canteras and Goto 1999b). More work is needed to rectify what role premammillary dorsal nucleus projections play in AHA regulation given that lesion of the premammillary dorsal nucleus does not significantly change predator-induced FOS expression in the AHA (Cezario et al. 2008). It is possible that these circuits are sufficiently redundant that lesion studies were not driving detectable effects using FOS methods with limited temporal resolution. The AHA may also receive a few scant synaptic projections from the parabrachial area according to Phaseolus vulgaris leucoagglutinin (PHA-L) anterograde tracing that were reported as camera lucida drawings (Bester et al. 1997). PHA-L tracing from the lateral hypothalamic area juxtaventromedial zone also indicates projections to the AHA (Hahn and Swanson 2015).

## Efferent projections of the AHA

Early cross-species work demonstrated short efferent connections from the AHA to the preoptic area, lateral hypothalamus, periventricular nucleus, dorsomedial nucleus, and to the capsule of the ventromedial nucleus as well as ranged projections to the lateral septal nucleus, dorsal premammillary nucleus, posterior hypothalamic area, and the central gray (Saper et al. 1978). This was corroborated by a report that employed Phaseolus vulgaris leucoagglutinin (PHA-L) staining to show that axons from the AHA follow three major routes. This includes a large ascending pathway that leads to the lateral septal nucleus and hippocampus, a second pathway that travels to the perifornical area of the lateral hypothalamus, ventrolateral tip of the nucleus reuniens, thalamus and habenula, as well as a third pathway that travels through the medial hypothalamic zone before ending in the periaqueductal gray (Risold et al. 1994). Much of this evidence was verified by secondary methods. For example, fluorogold retrograde tracing of the dorsomedial hypothalamus causes back-filled cells in the anterior hypothalamic area (Thompson and Swanson 1998). Lateral hypothalamic juxtaventromedial CTB injections result in back filling of the AHA, indicating that the AHA sends projections to it^28^. Using an adeno-associated viral (AAV) strategy that localizes enhanced green fluorescent protein (EGFP) to AHA^VGAT^ pre-synaptic terminals shows dense projections in the septum, dorsal premammillary nucleus, and the lateral/ventrolateral periaqueductal gray (Xie et al. 2022). AAV driven expression of synaptophysin fused mCherry also showed that AHA^PV^ neurons send dense projections to the dorsal premammillary nucleus (Laing et al. 2023). Fluorogold retrograde tracing from the premammillary dorsal nucleus showed that approximately half of the AHA^PV^ cells were backfilled. This projection was verified by channelrhodopsin2 assisted circuit mapping to release glutamate on approximately half of the neurons there.

## AHA output control of peripheral physiology and behavior

Early work investigating activation of the medial hypothalamus via microinjection of kainate or electrical stimulation evoked flight behavior and FOS in prototypical avoidance brain structures (Silveira et al. 1995). Histological visualization of implanted electrical stimulator tract can be observed in proximity to the AHA and camera lucida drawings of FOS indicate the AHA was activated, though immunoreactive cells were found within a 0.5 mm radius of the injection which spans nearly to the outer boundary of the hypothalamus. More modern seminal work showed that optogenetic stimulation of ventromedial hypothalamic terminals onto the AHA triggers avoidance and jumping behavior, which is consistent with the sympathetic activation that was observed upon ventromedial hypothalamic soma stimulation (Wang et al. 2015). Inhibition of the AHA mediated resulted in reduced corticosterone level increases after acute restraint stress, and disinhibition of the AHA results in a sustained increase in anxiety-like behavior following restraint stress (Bang et al. 2022b). These outputs contribute to context-directed behavior that is dependent on the environment (Bang et al. 2022a) and are likely modulated by septal inputs regulating stress induced anxiety behaviors (Anthony et al. 2014). Ensembles of AHA^VGAT^ neurons become activated during predator cue exposure, and their activity is strongly correlated with avoidance behavior during anxiety tests (Yan et al. 2022). Consistent with this finding, direct optogenetic activation of AHA^VGLUT2^ and AHA^CaMKIIa^ neurons resulted in jumping and running behaviors, while AHA^VGAT^ neurons significantly increased sniffing time (Hong et al. 2025). This coincides with findings that AHA^VGAT^ output to the lateral and ventrolateral periaqueductal gray results in decreased freezing behavior and risk assessment (sensory processing of environmental cues related to threat), while activation of their lateral septal outputs increases risk assessment and avoidance (Xie et al. 2022). Interestingly, AHA^PV^ neuron ablation is reported to alter responses during exposure to changes in environmental ambient temperature, whereby mice lacking AHA^PV^ neurons show reduced autonomic output, increases in blood pressure and heart rate (Mittag et al. 2013). This is consistent with the reported effect of losartan directly delivered to the AHA whereby it blocks production of catecholamines and changes in blood pressure (Kubo et al. 2001). While these studies provide compelling insight into the function of the AHA, the total repertoire of behaviors that result from AHA output is sufficiently broad that simultaneous acquisition of neural, behavior and physiological data in a variety of ethologically relevant environments is required to dissect mechanisms of control. The relationship between the AHA and physiological responses associated with fight-or-flight make a compelling case to test the effects on energy expenditure and feeding. The possibility of whether or not the AHA can suppress acute feeding behavior directly (as is seen with the premammillary dorsal nucleus (de Araujo Salgado and Krashes 2026)) is an important direction to explore. This would match the short term suppression of drinking behavior observed during activation of ventromedial hypothalamic projections to the AHA (Wang et al. 2015).

## Historical challenges

A major historical challenge for studying the anterior hypothalamic area can be traced to a simple semantic issue. Because the structure (anterior hypothalamic area) also corresponds to an anatomical direction (e.g. anterior), it is often grouped with other structures that are in the anterior segment of the hypothalamus – such as the suprachiasmatic nucleus, supraoptic nucleus, anterior periventricular nucleus, and the paraventricular nucleus (Xie and Dorsky 2017). Molecular commingling can also be attributed to shared developmental origins among cells of the anterior hypothalamus, which represents one of the four divisible regions of the hypothalamus based on RNA velocity trajectories and positional codes (Kim et al. 2020). This makes it challenging to label the AHA by a single molecular marker as was done with *NR5A1* (Bingham et al. 2006) and *SIM1* (Balthasar et al. 2005) for the ventromedial hypothalamus and paraventricular hypothalamus, respectively. While Foxd1 controls transcription markers *Six3* and *Vax1* in the anterior segment of the hypothalamus during development, these markers are not restricted to the AHA but rather attributable to the paraventricular nucleus, suprachiasmatic nucleus, and periventricular nucleus (Newman et al. 2018). Even rigorous modern transcriptomic work in the anterior portion of the hypothalamus often involves pooling of brain regions (Zhou et al. 2022). Across these studies, the challenge is ascribing the findings to the anterior hypothalamus which can be confused with the anterior hypothalamic area unless readers thoroughly check the details of each study.

This issue is compounded by difficult visualization of anatomical boundaries that often lead to mixing of the medial preoptic area with the AHA (Conrad and Pfaff 1975, Hurtazo et al. 2008, Paredes 2003). For example, Saper et al. (1978) sate that the region they identified as the AHA were evidently the medial preoptic area in Bleier’s (Fielding 1962) atlas (Bleier 1961). The poor separation of the AHA from adjacent areas like the medial preoptic area and lateral hypothalamus is conserved across species (Saper et al. 1978). This is critical for the use of techniques like in situ hybridization that would enable molecularly defined cell counts that characterize abundance in each parcel in the AHA. The lack of boundaries make it difficult to evaluate data from strategies that target drug delivery to the AHA by cannulation without any measure of containment of the drug (because most drugs cannot be visualized with microscopy) (Klir et al. 1994), even with very thorough and painstaking histological analysis (Falconi-Sobrinho et al. 2020). This is even more challenging when trying to interpret historical lesion studies (Olivier et al. 1983), which were foundational works that helped create the experimental paradigm for testing of defensive behaviors, but need to be evaluated in the context of knowledge obtained with more modern and precise methods.

Complete containment of injections to the AHA is an enormous challenge even with the most careful execution utilizing cutting edge surgical technology followed by painstaking histology. The scarcity of molecular markers that are physically distinct from neighboring regions makes genetically defined targeting of known cell types susceptible to the same issues. This has contributed to conflation in the scientific literature regarding the role of the AHA in aggression and mating which can only be clarified by cellular level experimental approaches, which show evidence against aggression dependent changes in AHA activity (Lin et al. 2011). The proximity of the AHA to adjacent areas makes it particularly difficult to isolate for exposure to circulating factors for the measurement of effects on behavior, physiology, or circuitry. Sophisticated combinations of slice electrophysiology experiments with synaptic blockades or the use of peptidergic bioluminescent sensors (Xia and Li 2025) are needed to measure direct effects on AHA activity.

In some human experiments, the medial preoptic area and AHA are operationalized as a single structure (Kang et al. 2021). For instance, one report demonstrated that cluster headaches are ascribed to differences in the anterior hypothalamus compared to patients with no headache, but the boundaries described as landmarks are for the anterior segment of the hypothalamus that is not specific to the AHA (Arkink et al. 2016). Human studies with functional brain imaging studies do provide intriguing insight into AHA function such as: (1) positively linked connectivity of the AHA with the anterior cingulate cortex, medial prefrontal cortex, and orbital frontal complex, (2) negatively linked connectivity of the AHA with the dorsolateral prefrontal cortex, the 13 L subdivision of the orbital frontal complex, and the insula while there is (3) mixed connectivity with the bed of the nucleus stria terminalis (Robertson et al. 2025). It is unclear how the efferent connections from the AHA contribute to the functional cortical changes that have been observed in human imaging studies which justifies future work for multi-node tracing using more modern techniques that combine retrograde expression of cre-recombinase with rabies tracing as has been done with other regions (Li et al. 2024). While functional imaging work in humans demonstrates the association of the AHA with many subregions of the brain, this approach lacks the resolution needed to dissect molecularly or functionally defined cell types for structures with heterogeneous activity patterns. It is likely that the ascending pathway from the AHA towards the hippocampus and habenula is the route of synaptic connectivity with the fewest intermediates. Functional imaging also brings limitations for definitive demonstration of causal directionality between structures. Thus, it is possible afferent inputs to the AHA give rise to the association. Though the work was not peer-reviewed, there are reports of a medial forebrain path infralimbic projection to the AHA in rats as well as peripeduncular area (Risold et al. 1997) which could contribute to the detection of associated activity (Brittain 1988). Of note, this projection was visualized at a later date as anterograde tracing from the caudal infralimbic cortex (Vertes 2004). Thus, human based approaches must continue to be advanced or combined with reverse translational approaches of animal models.

## Towards operationalization of the AHA

**Fig. 3:**
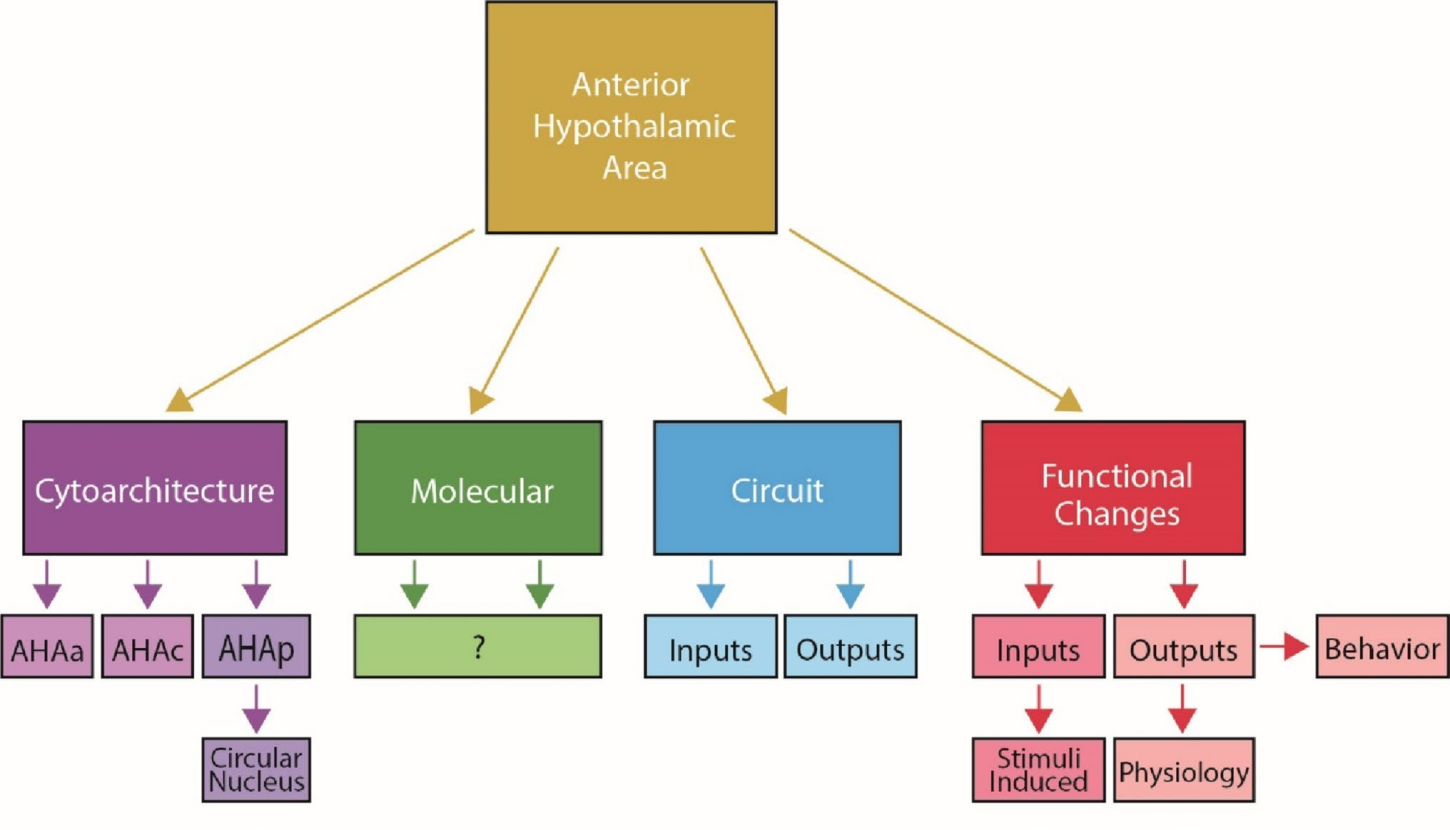
Schematic of cytoarchitectural, molecular, circuit, and functional approaches to operationalizing the AHA. The classic cytoarchitectural approach parcellates the AHA based on density of cell number. Molecularly defined populations of neurons that distinguish the AHA from neighboring regions or enable parcellation are not known. Circuit based approaches for afferent or efferent connections may designate individual parcels of the AHA but are not known. Finally, functional approaches related to the inputs (stimuli) or outputs (behavior and physiology) may define parcellations of the AHA

Numerous approaches may be useful in operationalizing the AHA (Fig. 3). The classic cytoarchitectural framework served as a starting point for investigation into the anterior hypothalamic area. It is evident that at least molecularly defined segments of the AHA exist as defined by the presence or absence of the AHA^PV^ neurons. In mouse brain, these neurons are situated in a tube between − 0.7 mm and − 1.2 mm posterior to bregma. At the anterior portion of the tube, they are sparsely located at the ventral region of the AHA, in the central portion of the tube they are densely clustered at the nucleus circularis located at the midline of the 3rd ventricle, and at the posterior portion of the tube they are sparsely located at the dorsal portion of the 3rd ventricle. Medial/laterally AHA^PV^ neurons are positioned between the fornix and the 3rd ventricle.

For the future of operationalization of AHA experimentation, it is critical to determine what if cytoarchitectural, molecular, circuit or functional definitions have the most utility. Part of the challenge for working in the anterior segment is that the boundary with the preoptic area is imperceptible (Saper et al. 1978) by classic cytoarchitectural methods. Indeed, early work by Risold, Canteras, and Swanson in the AHA recognized that this separation of anterior and AHA segments was possible by anatomical tracing of their outputs by carefully analyzing the effect of leaks to adjacent structures that vary across rodents (Risold et al. 1994). They note that their injections were confined “almost entirely” to the anterior hypothalamus but always contained cells outside the border. This tracing work needs to be followed up with channelrhodopsin2 assisted circuit mapping to identify which axons are fibers of passage, which form functional synapses onto targets, and what neurotransmitters are released. This foundational work using camera lucida drawings circumvented technical limitations with commendable transparent conservative reporting and serves as a useful starting point for mapping AHA output. The combination of tracers used and artistic renderings of biological structures justifies a push for modernizing this work. Consistent with this precedent, modern experimenters must leverage improved tools to employ a higher degree of rigor, disclose injection maps, and describe their outcomes with the highest degree of clarity possible.

It is critical for experimenters to define the parameters of their experiment so that other groups can interpret and extend findings. If it is an anatomical definition, rigid anatomical boundaries must be defined and evident in the data. To employ a circuit definition, anterograde/retrograde tracing is needed to distinguish synaptic inputs/outputs relative to the region of the anterior AHA, the AHC and AHP. If it is a molecular or cytoarchitectural definition, it is necessary to define hallmarks of population of neurons, channels, or receptors could be used to mark that zone. Notably, the tube that contains AHA^PV^ neurons is almost entirely vacant of other glutamatergic neurons and rich in GABAergic neurons (Laing et al. 2023). When employing a functional definition of the AHA, work must clearly demonstrate differences in behavioral/physiological functions between the regions tested. All of these approaches could be valid in different circumstances and it is not the place of this review to set bounds around a unanimous definition, but the precedent is to interpret boundaries conservatively with thorough injection controls (Saper et al. 1978).

**Table 1:** Chart of the species and sex in the reports cited

Citation (#)	Author	Species	Sex
[1]	Canteras et al. (2002)	-	-
[2]	Saper et al. (1978)	Cat, rat, and squirrel monkey	-
[3]	Saper et al. (1979)	Cat and squirrel monkey	-
[4]	Brown et al. (1999)	Mouse	Female and male
[5]	Wolfe et al. (2005)	Mouse	-
[6]	Laing et al. (2023)	Mouse and rat	Female and male
[7]	Hong et al. (2025)	Mouse and rat	Female and male
[8]	Xie et al. (2022)	Mouse and snake	Female and male
[9]	Harder et al. (2018)	Mouse	Female and male
[10]	Xu et al. (2022)	Mouse	-
[11]	Cullinan et al. (1995)	Rat	Male
[12]	Cezario et al. (2008)	Rat	Male
[13]	Martinez et al. (2008)	Mouse and rat	Male
[14]	Laing et al. (2025)	Mouse	Female and male
[15]	Mitra et al. (2016)	Rat	-
[16]	Torossian et al. (2025)	Mouse	Female and male
[17]	Mittag et al. (2013)	Mouse	Female and male
[18]	Machado et al. (2022)	Mouse	-
[19]	Jahan et al. (2015)	Mouse and rat	Female and male
[20]	Bartholomew et al. (1993)	Mouse	Female
[21]	Korneva et al. (2000)	Rat	Male
[22]	Hagiwara et al. (2005)	Rat	Male
[23]	Bang et al. (2022a)	Mouse	Female and male
[24]	Anthony et al. (2014)	Mouse	Male
[25]	Canteras et al. (1999a)	Rat	Male
[26]	Canteras et al. (1999b)	Rat	Male
[27]	Bester et al. (1997)	Rat	Male
[28]	Hahn et al. (2015)	Rat	Male
[29]	Risold et al. (1994)	Rat	Male
[30]	Thompson et al. (1998)	Rat	Male
[31]	Silveira et al. (1995)	Rat	Male
[32]	Wang et al. (2015)	Mouse	Female and male
[33]	Bang et al. (2022b)	Mouse	Male
[34]	Yan et al. (2022)	Mouse	Male
[35]	Kubo et al. (2001)	Rat	Male
[36]	de Araujo Salgado et al. (2026)	Mouse	Female and male
[37]	Xie et al. (2017)	-	-
[38]	Kim et al. (2020)	Mouse	Female and male
[39]	Bingham et al. (2006)	Mouse	-
[40	Balthasar et al. (2005)	Mouse	Female and male
[41]	Newman et al. (2018)	Mouse	-
[42]	Zhou et al. (2022)	Mouse	Female
[43]	Conrad et al. (1975)	Rat	Female and male
[44]	Hurtazo et al. (2008)	Rat	Male
[45]	Paredes (2003)	Rat	Male
[46]	Fielding (1962)	-	-
[47]	Bleier (1961)	Cat	-
[48]	Klir et al. (1994)	Rat	Male
[49]	Falconi-Sobrinho et al. (2020)	Mouse	Male
[50]	Olivier et al. (1983)	Rat	Male
[51]	Lin et al. (2011)	Mouse	Female and male
[52]	Xia et al. (2025)	HEK293T cells	-
[53]	Kang et al. (2021)	Human	-
[54]	Arkink et al. (2016)	Human	-
[55]	Robertson et al. (2025)	Human	-
[56	Li et al. (2024)	Rat	-
[57]	Risold et al. (1997)	-	-
[58]	Brittain (1988)	Rat	-
[59]	Vertes (2004)	Rat	Male

Rather than prescribe a definition to the scientific community, this review serves to highlight the importance of clear operationalization within each manuscript. Especially cautious and thorough histological analysis must be conducted to allow readers to understand the relationship between manipulations, outcome measures, and author interpretations. This will be essential for any future synthesis of studies that are conducted between groups and across time. Continued synthesis will be necessary to rectify discrepancies in AHA regulation, output functions, and relevance to mental health disorders. Much of the work in the AHA has been conducted primarily in rodent (52 of the 61 AHA studies cited here) so future studies should expand into other model organisms. In addition, the majority of studies reviewed here are on male only (22 of 40 that specify) with 16 using both sexes and only 2 using females only while 18 do not specify. Even in the studies that have both sexes, they were not necessarily powered to detect sex differences and this may be an important consideration for future studies. While it is increasingly understood that the AHA is a neuronal structure tuned to respond to threats via inputs that are layered across timescales (synaptic and circulating factors) for the promotion of survival (behavioral and physiological functions), more work needs to be done in disease models to understand how AHA activity patterns influence symptomology.

## Data Availability

No datasets were generated or analysed during the current study.
